# Transcriptomic Profiles in Zebrafish Liver Permit the Discrimination of Surface Water with Pollution Gradient and Different Discharges

**DOI:** 10.3390/ijerph15081648

**Published:** 2018-08-03

**Authors:** Zhou Zhang, Wei Liu, Yuanyuan Qu, Xie Quan, Ping Zeng, Mengchang He, Yanmei Zhou, Ruixia Liu

**Affiliations:** 1 Key Laboratory of Industrial Ecology and Environmental Engineering (MOE), School of Environmental Science and Technology, Dalian University of Technology, Dalian 116024, China; andar2008@126.com (Z.Z.); qyy007@126.com (Y.Q.); quanxie@dlut.edu.cn (X.Q.); 2 State Key Laboratory of Environmental Criteria and Risk Assessment, Chinese Research Academy of Environmental Science, Beijing 100012, China; zengping@craes.org.cn (P.Z.); liurx@craes.org.cn (R.L.); 3 State Key Laboratory of Water Environment Simulation, School of Environment, Beijing Normal University, Beijing 100875, China; hemc@bnu.edu.cn; 4 Department of Civil and Environmental Engineering, Beijing Key Laboratory of Aqueous Typical Pollutants Control and Water Quality Safeguard, Beijing Jiaotong University, Beijing 100044, China; zym721101@163.com

**Keywords:** zebrafish, microarray, gene expression, pathway enrichment, endocrine disruption, human disease

## Abstract

The present study aims to evaluate the potential of transcriptomic profiles in evaluating the impacts of complex mixtures of pollutants at environmentally relevant concentrations on aquatic vertebrates. The changes in gene expression were determined using microarray in the liver of male zebrafish (*Danio rerio*) exposed to surface water collected from selected locations on the Hun River, China. The numbers of differentially expressed genes (DEGs) in each treatment ranged from 728 to 3292, which were positively correlated with chemical oxygen demand (COD). Predominant transcriptomic responses included peroxisome proliferator-activated receptors (PPAR) signaling and steroid biosynthesis. Key pathways in immune system were also affected. Notably, two human diseases related pathways, insulin resistance and *Salmonella* infection were enriched. Clustering analysis and principle component analysis with DEGs differentiated the upstream and downstream site of Shenyang City, and the mainstream and the tributary sites near the junction. Comparison the gene expression profiles of zebrafish exposed to river surface water with those to individual chemicals found higher similarity of the river water with estradiol than several other organic pollutants and metals. Results suggested that the transcriptomic profiles of zebrafish is promising in differentiating surface water with pollution gradient and different discharges and in providing valuable information to support discharge management.

## 1. Introduction

Whether toxicity test results can reflect responses in aquatic vertebrates stressed by actual aquatic environmental pollution remains a key issue. Many aquatic environments receive large amounts of contaminants from human activities, even after wastewater treatment. The environmental transformation of chemicals likely forms products that retain bioactive moieties, sometimes exhibiting higher toxicity than the parent compounds [1,2,3]. Exclusive physicochemical parameters and targeted chemical analysis are insufficient to evaluate the potential integrated biological effects [4]. While simple bioanalytical methods provide efficient surrogate monitoring tools for water quality determination, conventional toxicity tests are limited due to the selected endpoints [5].

Toxicogenomic approaches are promising tools to remotely evaluate the toxicity of environmental samples by providing gene expression information. These genomic tools may provide evidence about the mechanisms and modes of action (MOA) for classes of chemicals, enabling the extrapolation from responses at the individual level to effects on populations, and to define species differences in toxicity [6]. Particularly, the highly chemical-specific gene signatures prompt the use of toxicogenomic tools for identification of pollutants classes and pollution source in environmental monitoring and diagnosis. Yang et al. [7] recognized chemical-specific genomic profiles in barcode-like patterns in zebrafish embryos, enabling the discrimination of individual compound based on gene expression changes. It would profit greatly to provide guidance of what chemicals could be identified as major drivers for organisms affected by exposure to the chemical mixtures present in aquatic effluents.

With the rapid growth of using transcriptomic analyses in aquatic toxicology, its application in chemical mixtures commonly encountered in the field is still in the early stages. We are facing many challenges in understanding the big data obtained using this powerful tool, while researchers have successfully made some progresses to use genomic profiles of aquatic organisms exposed to wastewater effluent [8,9,10,11,12] and surface water [13,14]. Falciani et al. [15] used gene expression fingerprints to distinguish the origins of flounder taken from sites of different pollution status. Martinovic-Weigelt et al. [12] found that transcriptomic analysis in fathead minnow liver was capable of distinguishing wastewater treatment plant effluent, upstream and downstream ambient water.

The present study is part of a National Water Pollution Control and Management Project conducted in the Liao River basin of China. In other parts of the project, the chemical analysis of target compounds and the source to sink analysis were conducted [16,17,18]. The present study focused on analyzing transcriptomic profiles in zebrafish exposed to the surface water, with the aim to evaluate the impacts of complex mixtures of pollutants at environmentally relevant concentrations on aquatic vertebrates, as well as to elucidate the potential of transcriptomic profiles to differentiate pollution sources, and to identify the toxic components. The Hun River is a main tributary located in the Liao River basin, flowing through the city of Shenyang which is the capital of Liaoning Province and one of the most important industrial cities in China [16,19]. The Dongzhou River and Xi River, two main tributary flowing into Hun River, have been identified as the receiving water of chemical wastewater and pharmaceutical wastewater, respectively [20,21]. Hence, the selected sampling area provided a good model for comparison of the transcriptomic profiles in surface water across pollution gradients and receiving different discharges. The sampling sites were selected at the upstream and downstream of Shenyang City, and the mainstream and tributary near the junction. Zebrafish (*Danio rerio*) microarrays combined with bioinformatic analysis were employed to compare the transcriptomic profiles among sites. Furthermore, the gene expression profiles of zebrafish exposed to river water were compared with those of zebrafish exposed to single chemical from the literature. Genome expression profiles were characterized for livers because the organ integrates xenobiotics from multiple routes of entry (gill, intestine, and skin), is the primary tissue for their metabolism.

## 2. Methods and Materials

### 2.1. Site Selection and Water Sampling

**Figure 1:**
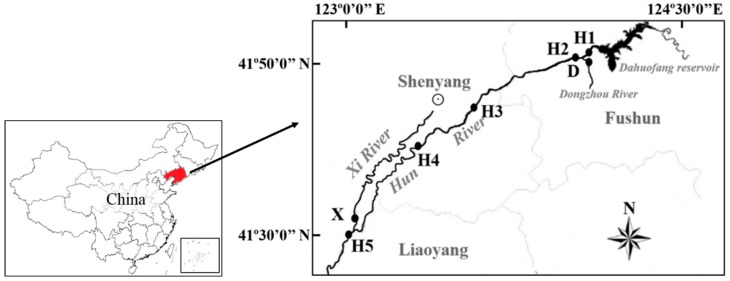
Locations of the sampling sites.

Five sampling sites were located along the Hun River and one site each was on the Dongzhou River and the Xi River (Figure 1). The Dongzhou River is a tributary located at the upper reach of the Hun River, mainly receiving the discharges from petroleum and chemical industries [20]. The Xi River flows into the lower reach of Hun River, receiving large scale domestic and industrial effluent from the pharmaceutical industry [21].

In September, 2014 grab samples were collected in 20 L clean polypropylene bottles which were pre-rinsed with river water three times before sample collection. Tap water aerated for more than 48 h and filtered through activated carbon (1 mm, Huaiyushan Activated Carbon Co. Ltd., Zhuhai, China) was also used in the field as procedural control. The collected water samples were brought back to the laboratory in a cooler and stored at 4 °C for bioassay within 48 h. All samples were analyzed for the following parameters: dissolved oxygen (DO), pH, chemical oxygen demand (COD) and ammonia nitrogen (NH_3_-N). DO and pH were measured immediately after sampling by DO analyzer (Mettler-Toledo International Inc., Columbus, OH, USA) and pH analyzer (Sartorius, PB-20, Göttingen, Germany). After the surface water was filtered with glass fiber filter (GF/B, Whatman, Maidstone, UK), COD was measured by the microwave-assisted potassium dichromate method [22], and ammonia nitrogen was determined using Nessler’s reagent spectrophotometric method [23]. Descriptions of these sampling sites, as well as the physicochemical water parameters, are given in Table 1. Eighty two organic chemicals, including phthalic acid esters (PAEs), polycyclic aromatic hydrocarbons (PAHs), benzene hydrocarbons and phenolic compounds, were quantitatively analyzed by gas chromatography-mass spectrography conducted in other parts of the project (National Water Pollution Control and Management Project conducted in the Liao River basin of China). Water samples of sites H5 and X were not included in the chemical analysis. A detailed description of the chemical analysis is presented in the Supporting Information.

### 2.2. Fish Exposures

Adult wild-type zebrafish (*Danio rerio*) were purchased from a local commercial distributor, and were maintained in culture tank at 27 ± 1 °C with a 14:10 h light/dark photoperiod for acclimatization for two weeks. The culture water was aerated at least 24 h, and the fish were fed twice daily with commercial food (Guangzhou Fenglaideng Trading Co., Ltd., Guangzhou, China). The mortality rate of zebrafish was less than 5% during acclimatization. Male zebrafish were selected for exposure, and confirmed by the observation of gonads during anatomy. The gender difference was not included into the design of the study.

River water samples were filtered with glass fiber filter (GF/B, Whatman) to avoid the bias caused by the suspended substances to the toxicity test [24]. Dissolved oxygen concentration (>6 mg/L) and pH (7–8.5) were measured to ensure the water quality before the exposure. Each tank contained 1.5 L water and 5 male zebrafish, and static exposure was conducted for 96 h. No fish deaths occurred during the exposure. Fish were anesthetized by incubation in ice water after exposure [25] and the livers were surgically collected for total RNA isolating. All experimental procedures were approved by the Ethics Committee, Dalian University of Technology, China (No. 2018-035).

### 2.3. Microarray Analysis

Pooled liver tissues from five fish were used for RNA extraction. Total RNA was extracted using TRIZOL Reagent (Life Technologies, Carlsbad, CA, USA), and purified by RNeasy mini kit and RNase-Free DNase Set (Qiagen GmBH, Hilden, Germany) following the manufacturer’s instructions. RNA integrity was checked by a Bioanalyzer 2100 instrument (Agilent Technologies, Santa Clara, CA, USA). The quality control criteria of RNA samples including RNA integrity number (RIN) value ≥7.0, 28S/18S > 0.7 and value of A260/A280 between 1.9 and 2. Total RNA samples were stored at −80 °C and were sent to CapitalBio Corporation (Beijing, China) for microarray analysis. Two independent microarray experiments for each sample were performed with Agilent Zebrafish Oligo Microarray (V2) (4 × 44 K) which contains 43,803 probes representing 23,207 gene according to the manufacturer’s instructions. Briefly, 2 μg total RNA was amplified and labeled by Low Input Quick Amp Labeling Kit, One-Color (Agilent). Each slide was hybridized with 1.65 μg labeled RNA using Gene Expression Hybridization Kit (Agilent) in a hybridization oven (Agilent). Slides were washed in staining dishes (Agilent) after 17 h hybridization. The signal was obtained by using the Agilent Microarray Scanner (Agilent) with default settings. Raw data were normalized by the Quantile algorithm using GeneSpring Software 13.0 (Agilent). The microarray data from this study were deposited with the Gene Expression Omnibus repository (GSE92330 and GSE108227). The determination of differentially expressed genes (DEGs) were implemented in GeneSpring Software 13.0 based on fold change (FC) value compared with control. For each comparison, DEGs obtained as genes with a “detected” call in one or both populations were selected. Only genes that showed FC ≥ 2 in both two independent microarray analysis were sent for further pathway enrichment analysis, clustering and principal components analysis (PCA). For site D, because one replicated RNA sample was not qualified in the determination of RNA quality, only one microarray analysis was conducted and DEGs were identified by FC ≥ 3.

### 2.4. Pathway Analysis

Database for Annotation, Visualization and Integrated Discovery (DAVID version 6.8) [26] was used to enrich biological pathways from the DEGs that could be mapped to Kyoto Encyclopedia of Genes and Genomes (KEGG) [27] pathway orthologs. *p*-values calculated in DAVID was used to examine the significance of gene-term enrichment with a modified Fisher’s exact test [26]. For individual pathways, significant enrichment was considered if *p* < 0.05, and more than two DEGs were associated with the pathway [13].

### 2.5. Clustering Analysis

The microarray data were Log2 transformed and median centered by samples using the Adjust Data function of CLUSTER 3.0 software [28], and then further analyzed with hierarchical clustering with average linkage. All DEGs in zebrafish exposed to river water from each site were included in the hierarchical clustering analysis, and tree visualization was performed using Java Treeview (Stanford University School of Medicine, Stanford, CA, USA).

Clustering analysis was also used to compare the gene expression profiles of zebrafish exposed to the river water and that to single model chemical. The microarray data of zebrafish exposed to estradiol (E2, 50 μg/L,GSE30050), nitrophenol (NP, 30 μg/L,GSE30058), arsenic [29] (As, 192 μmol/L, GSE3048), benzo-[A]-pyrene (BAP, 5 μg/L, GSE30035), cadmium [30] (Cd, 30 μg/L,GSE41623), and chloroaniline (CA, 30 μg/L, GSE30055), were downloaded from NCBI GEO database (http://www.ncbi.nlm.nih.gov/geo/). Although the concentrations of these chemicals employed in the zebrafish exposure research were higher than detected concentrations in the river water, many other compounds coexisted in the surface water, which affected aquatic vertebrate in similar mode with these typical pollutants. Hence, it is reasonable to compare the gene profiles in the zebrafish exposed to single compound and those exposed to the surface water [8]. These model chemicals were selected because of the typical chemical structure, toxic mechanism, as well as the data availability. For example, E2 was selected for comparison to indicate the potential endocrine disruption effects. Microarray data generated by liver of adult male zebrafish with 96 h exposure were selected from the GEO database. In order to compare the gene expression profiles obtained from different microarray platforms, the probe IDs were transformed to gene symbols, and the fold change of each gene, as well as *p* values (*t*-test), were calculated by the normalized signal values with Excel (Microsoft Office 2013, Microsoft Corporation, Redmond, WA, USA). DEGs with *p* < 0.05 in zebrafish exposed to the model chemicals were compared by clustering analysis to those exposed to the river surface water.

### 2.6. Statistical Analysis

Statistical analysis of the correlation between DEGs number and COD, NH_3_-N, DO and pH was performed with SPSS v16.0 (SPSS Inc., Chicago, IL, USA) and the differences were considered statistically significant when *p* value was less than 0.05 using the Pearson’s correlation coefficient. The PCA for experimental data were performed using the princomp function of the R statistical package [31] (R version 3.3.3 2017, http://www.R-project.org).

## 3. Results

### 3.1. Differentially Expressed Genes Affected by River Water

**Figure 2:**
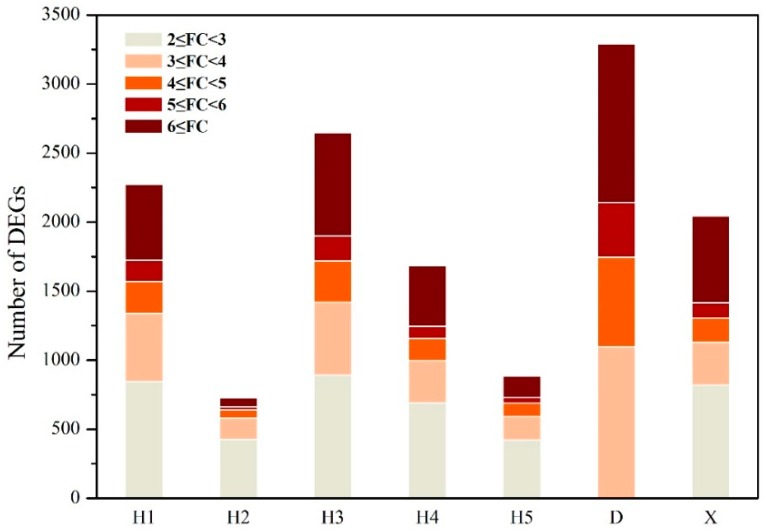
Distribution of fold change of differentially expressed genes in zebrafish liver exposed to surface water. The fold changes were average data of the two independent microarray analysis. Bars with different color represent FC value range. Grey bars represent total DEGs number of different samples.

The number of DEGs in the livers of zebrafish exposed to surface water from the seven sites ranged from 3292 at site D (Dongzhou River water) to 728 at site H2 (Hun River water at the downstream of the junction of the Dongzhou River and the Hun River) (Table S1). The number of DEGs was positively correlated with COD of river water (*r* = 0.788, *p* = 0.035, Table S2). The variations in the expression level of the DEGs were mainly in the range of 2~3 fold, accounting for 39~59% of total DEGs (Figure 2). 

There were 10~34% of DEGs in each treatment with fold change value higher than 6. The distributions of DEGs with different fold change value range were similar between different sites.

### 3.2. Enriched Pathway of DEGs Affected by River Water

**Figure 3:**
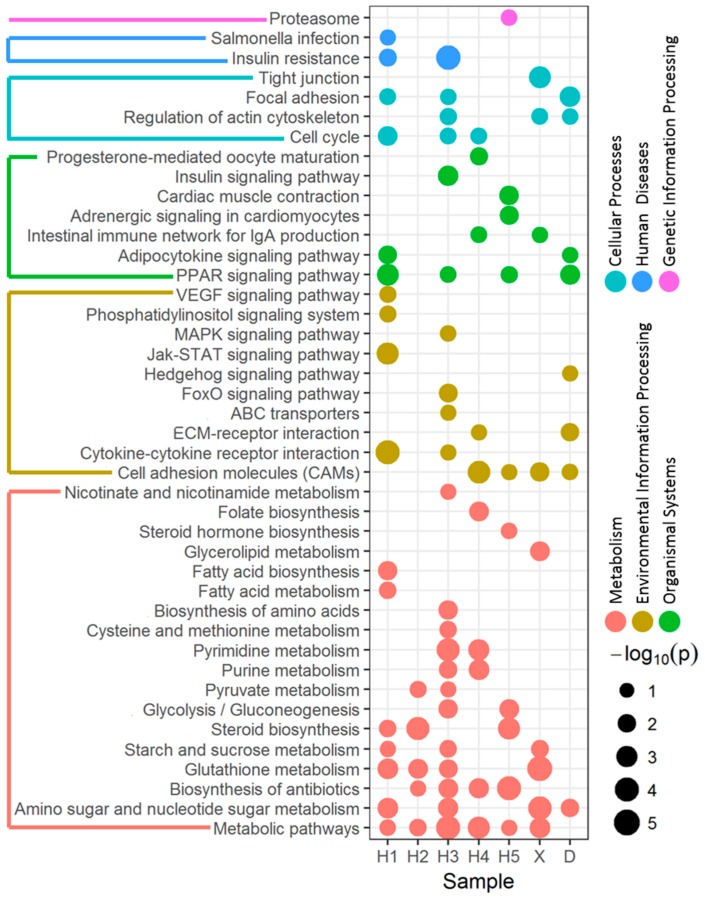
Pathway enrichment analysis of DEGs in liver of zebrafish exposed to surface water. The area of circle represents −log_10_(p), that bigger circles have lower p values. The color of circles indicates functional categories of pathways.

Functional pathway enrichment analysis was conducted to develop an understanding of biological implications of the DEGs in each site based on the KEGG database. Overall, gene expression in 17, 5, 22, 10, 10, 9 and 8 pathways was significantly enriched in zebrafish exposed to water from the H1, H2, H3, H4, H5, X and D sites, respectively. The *p* values and functional categories of pathways of each site were shown in Figure 3.

Gene expression in metabolic pathways responsible for amino sugar and nucleotide sugar, glutathione, starch and sucrose, steroid, glycolysis/gluconeogenesis, purine as well as pyrimidine was affected by at least two of the seven surface water samples. Each water sample induced DEG expression in 5–12 metabolic pathways, except that site D only affected 1 metabolic pathway. These pathways were associated with carbohydrate metabolism, lipid metabolism and nucleotide metabolism. Main DEGs involved in these metabolic pathways are those of cytochrome P450 family members, such as *cyp51*, *cyp11c1*, *cyp17a1* and *cyp19a1b*. These genes were differentially expressed in more than three surface water samples, and *cyp51* was affected by five samples.

Effects on the endocrine system were highlighted through the microarray assessments. Gene expression in the peroxisome proliferator-activated receptors (PPAR) signaling pathway was affected by surface water from site H1, H3, H5 and D. Several PPAR-regulated genes were altered, such as those coding for acyl-CoA synthetase genes that are key enzymes involved in the initial steps of fatty acids metabolism. In addition, the expression of genes in the steroid biosynthesis pathway was affected by surface water from sites H1, H2 and H5. Several enzymes involved in the steroid hormone biosynthesis pathway are recognized as important targets for endocrine-disrupting chemicals. For example, the activity of the cytochrome P450 enzyme *cyp51* whose expression was altered by five surface water samples, is inhibited by some azole fungicides and antifungal drugs, resulting in the blockage of ergosterol biosynthesis [32]. Steroid hormone biosynthesis pathway expression was enriched for site H5 with the upregulation of *cyp17a1* and hydroxysteroid (17-beta) dehydrogenase 1 (*hsd17b1*) and the downregulation of *cyp11c1*.

Several other affected functions were suggested by the enriched pathway. These included cellular processes such as cellular community (focal adhesion and tight junction), cell growth and death (cell cycle) and cellular motility (regulation of actin cytoskeleton), which were induced by surface water from sites H1, H3, H4, X and D. Growth arrest and DNA-damage-inducible related genes (*gadd45aa* and *gadd45bb*) enriched in cell cycle pathway were upregulated by site H1 and H3 water, while site H4 water upregulated the expression of cell division cycle related genes (*cdc6*, *cdc7* and *cdc20*) in cell cycle pathway. Enriched expression of the proteasome pathway assigned to genetic information processing was detected in the livers of zebrafish exposed to surface water from site H5. The site H5 water upregulated the expression of three subtypes of proteasome subunit (*psmb7*, *psmb8f* and *psmb9b*) and three subtypes of proteasome 26S subunit (*psmd1*, *psmd4a* and *psmd11b*).

Potential immunotoxic effects were revealed by interference in gene expression in pathways associated with intestinal immune network for IgA production induced by site H4 and X water. For example, expression of chemokine ligand 12b (*cxcl12b*) enriched in this pathway was upregulated by site H4 water but downregulated by site X water, and heat shock protein (*hsp90aa1.2*) was upregulated in the zebrafish exposed to H4. The expression of genes in pathways related to circulatory system such as adrenergic signaling in cardiomyocytes and cardiac muscle contraction were affected by site H5 water, such as the upregulation of ATPase-related genes (*atp1a3b* and *1tm1b2a*) and troponin related genes (*tnnc1a* and *tnnc1b*).

Particularly, insulin resistance and salmonella infection pathways, which were related to human disease, were affected by sites H1 and H3. Interleukin related (*il1b*) and chemokine ligand 8a (*cxcl8a*) gene expression was upregulated by site H1, which were enriched in salmonella infection pathway. Preproinsulin (*ins*) was down-regulated by both site H1 and H3 water and thus could affect the function of the insulin resistance pathway.

### 3.3. Differentiation of Gene Profiles for Pollution Gradients and Discharge Source

**Figure 4:**
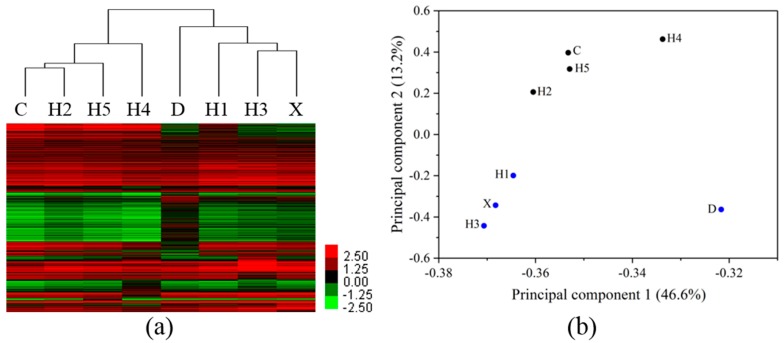
Hierarchical clustering (**a**) and principal component analysis (**b**) of differentially expressed genes in the liver of zebrafish exposed to river water of each site. Upregulated genes are marked in red and downregulated genes in green. Scale shows fold differences in expression versus controls (**a**).

Clustering analysis resulted in two distinct clusters corresponding to the selected sites (Figure 4a). When the gene profiles of zebrafish liver exposed to the water sample from tributary and the mainstream near the junction were compared, it was found that site X and site H5 were assigned to two groups with the lowest similarity. Site D and H2 were also assigned to two groups. Site H3 and H4, the upstream and downstream of Shenyang City, were separated in two groups. The PCA of DEGs showed the significant differences in PC scores occurring within the first two principal components (PC1 and PC2, 59.8%; Figure 4b). The seven sampling sites were separated along PC2. A relatively higher difference of gene expression profiles between site X and H5 along PC1 was also observed. Sites H3 and H4 were separated along PC2. Differences of gene responses between downstream (H4) and upstream (H3) of Shenyang City, and between mainstream (H2, H5) and tributary sites near the junction (D, X) were compared (Figure S1). The oppositely changed genes accounted for 1–8% of the total DEGs for each site, while the commonly changed genes accounted for 9.5–29% of the total DEGs. The PCA results were consistent with the clustering analysis results, further supporting the difference in the transcriptomic profiles between the tributary and the mainstream, and that between the upstream and downstream of the Shenyang City.

**Figure 5:**
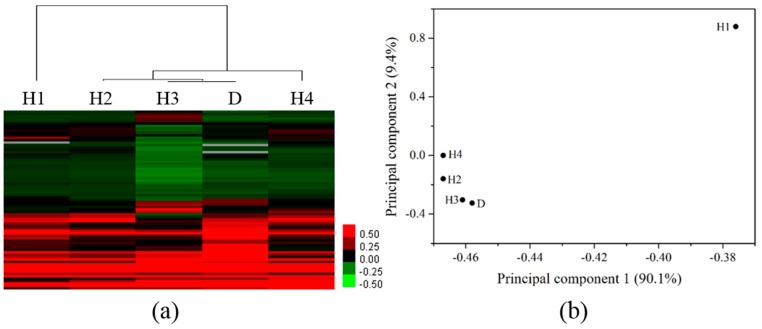
Hierarchical clustering (**a**) and principal component analysis (**b**) of chemicals detected in the surface water from the Hun River. 82 chemicals were used for clustering analysis and PCA. Scale shows fold differences in expression versus controls (**a**).

A total of 82 organic chemicals, which are typical pollutants discharged from industrial activities in Hun River basin, such as oil refineries, plastic plants, dyes, pesticides, pharmaceuticals, coal conversion, coking and petrochemical, were detected in the surface water from H1, H2, H3, H4 and D (Table S3). Correlation analysis between DEGs number and concentrations of these chemicals found that only two chemicals concentrations were significantly correlated (*p* < 0.05) with DEGs number. Eleven chemicals that concentrations were commonly at top 20 in all samples were listed in Table 2. Diisobutyl phthalate was the most abundant chemical, with concentrations of 5.03~125 μg/L, and the highest concentration was detected at site D. Besides, 26 of 82 chemicals, mainly including PAEs, phenolic and heterocyclic compounds, were detected at higher concentrations at site D than those at the other 4 sites. Clustering analysis conducted with the detected chemicals resulted in the separation of H1 from other sites, because H1 was a background site near the reservoir (Figure 5a). The PCA of detected chemicals in the surface water samples were consistent with the clustering analysis, which showed obvious separation with the majority of the variation (99.5%, Figure 5b).

When gene expression profiles of zebrafish exposed to surface water were compared with that of zebrafish exposed to selected chemicals, no obvious grouping was obtained when the average results from two independent microarray analysis were used for analysis. However, one of the microarray data found that the expression profiles of fish exposed to surface water from each site were very similar to those of fish exposed to E2 (Figure S2). The clustering analysis suggested relatively higher similarity of the gene profiles in the zebrafish exposed to surface water with that exposed to E2, than the other several organic compounds and metal, providing another line of evidence for the potential endocrine disruption effects.

## 4. Discussion

Toxicogenomic studies have been mainly used to determine genomic responses and to analyze toxicity pathways of individual chemicals. However, a few studies [12,13,33] have applied transcriptomics-based methods to complex environmental samples. The present study evaluated the feasibility of using zebrafish microarrays as a biomonitoring tool to assess the potential hazardous effects in aquatic organisms exposed to pollutants in surface water, and its potential in discriminating pollution gradient and different discharges.

The significantly positive correlation between DEG number and COD (Table S2) suggested that the number of DEGs may be indicative of the pollution level of the surface water. The number of altered genes in the D site was the highest among the 7 sites, consistent with the chemical analysis results that concentrations of the 82 selected chemicals were higher at site D. However, there was no significant correlation between DEG number and concentrations of any chemicals. A possible reason is that toxicological changes reflect the combined effects of various kinds of chemicals, rather than any specific compound. Yang et al. [7] have found significant positive correlations between the number of altered genes in zebrafish embryos with the concentrations of cadmium chloride, 1,1-bis-(4-chlorophenyl)2,2,2-trichloroethane (DDT), 2,3,7,8-tetrachlorodibenzo-*p*-dioxin (TCDD), valproic acid, 4-chloroaniline, and methylmercury chloride. The number DEGs in human hepatoma HepG2 cells induced by exposure to aquatic effluents were significantly decreased [34]. However the H1 site water, a background sample collected from the reservoir, also affected the expression of more than 2000 genes. Therefore, DEG numbers induced in zebrafish could be used as a reference parameter when evaluating toxicity based on genomics analysis. However, it is suggested that number of DEGs is to be used together with the function and pathway analysis instead of being used alone when evaluating the toxic potential of the surface water.

The gene profiles of zebrafish exposed to surface water suggested transcriptomic changes related to endocrine disruption. The DEGs and pathway enrichment highlighted the disturbance in endocrine disruption. Furthermore, insulin resistance was enriched, which was classified as pathway related to human endocrine and metabolic diseases. Moreover, the enrichment of PPAR signaling pathway and concomitant detection of phthalates in the river water samples were consistent with results of a number of studies in fish that phthalates induced PPAR activation [35]. Previous studies have found the occurrence of intersex fish [36] and increasing expression of vitellogenin (*vtg*) [37] in wild crucian carp sampled from the Hun River. The present study provided molecular evidence for the observed reproductive abnormalities in the field.

The expression of genes in pathways related to cell cycle growth arrest and genetic information processing was also affected, suggesting that the surface water potentially induced genetic damage. Cell cycle growth arrest is an important cellular response to genotoxic stress, and *gadd45* enriched in cell cycle pathway which was altered by water from sites H1, H3 and H4, was regulated by p53 stress protein and plays an important role in the cycle G2-M checkpoint [38]. Cell division cycle has a critical role in the initiation of DNA replication, and *cdc6* which was deregulated by sites H1 and H4 has been reported to increase the replication activity with subsequent DNA damage [39]. The proteasome affected by site H5 is related to folding, sorting and degradation of protein. Proteasome subunits (*psmb7*) whose expression was upregulated by site H1 water, was induced only in the liver and regulated by zebrafish Nrf2 which has a trypsin-like protease activity [40]. Elevated proteasome expression has been suggested as an indicator of exposure to metal ions [41].

Besides the endocrine disruption and genetic damage, which are the main toxic responses employed in the conventional endpoint based toxicity test of environmental water samples, the transcriptomic analysis also suggested that these may by alteration in essential biological functions such as immune system and circulation system. No validated bioassays exist to test such adverse effects. *Cxcl12b* enriched in the intestinal immune network for IgA production pathway was originally identified in the immune system, which plays crucial roles in leukocyte trafficking and immune responses [42]. Increased level of *hsp90aa1.2* was observed in zebrafish exposed to site H4 water, consistent with the results that the expression levels of various *hsp* genes are increased in fish exposed to environmental pollutants, such as microcystin-LR [43], heavy metals [44] and industrial effluents [45]. It was reported that silica nanoparticles induced cardiac dysfunction of zebrafish embryo and inhibited the cardiac muscle contraction via affecting related genes, such as ATPase-related genes and regulatory gene *tnnc1a* for cardiac troponin C [46], and these genes were also altered in the liver of zebrafish exposed to the sampled Hun River water.

The expression of genes related to human disease pathways were affected by site H1 and H3 water, and indicated potential adverse effects in humans. Comparison with the human reference genome showed that 71.4% of human genes haves at least one obvious zebrafish orthologue and 69% of zebrafish genes have at least one human orthologue [47]. Hill et al. [48] suggested that the function and structure of adult zebrafish liver is similar to the mammalian liver, and there is also high genetic conservation between zebrafish and humans. Besides, immune system of zebrafish has proven to be significantly similar to that of humans [49]. Insulin resistance plays a central role in type 2 diabetes, obesity, and metabolic syndrome, which are pathologies affecting a large population worldwide. Zebrafish appeared to be sensitive to human insulin and turn to be insulin resistant when exposed to high dose of the hormone [50]. *Cxcl8* is expressed in leukocytes of zebrafish as in humans, and is upregulated under inflammatory conditions induced by chemical or bacterial insult [51]. However, to analyze the correlation between gene expression and human disease pathway need to measure insulin resistant related endpoints.

The gene expression profiles in zebrafish exposed to the surface water samples collected from the mainstreams were differentiated from the tributaries near the junction, and also discriminated between the upstream and downstream water of Shenyang. In contrast, the chemical profiles based on the data of 82 typical organic pollutants analysis were only capable of differentiating H1, the background sample, from the other surface water samples. The results suggested that transcriptomic profiles of the surface water were highly specific despite of the similar pollutant profiles in adjacent sample sites. Falciani et al. [15] found that the gene profiles in flounder could be well identified from a highly polluted area, but flounder from other areas were not perfectly segregated by clustering analysis. Gene profiles in aquatic species have been used to evaluate the contribution of effluent from wastewater treatment to the downstream pollution [12,33]. The results of the present study suggested that transcriptomic tools have the potential to discriminate the pollution profile of surface water with adjacent spatial position and overlapped pollutants profile.

The identification of pathways related to endocrine disruption, as well as the similarity of gene expression profiles of zebrafish exposed to surface water and E2, indicating that the occurrence of endocrine disrupting chemicals. Several endocrine disruption chemicals such as diisobutyl phthalate (DIBP), di(2-ethylhexyl) phthalate (DEHP), dibutyl phthalate (DBP) and dimethyl phthalate (DMP) were detected in the surface water with their concentrations up to 125, 11, 3, and 1 μg/L. The median lethal concentrations (LC50) of fish for 96 h exposure to DIBP, DEHP, DBP and DMP were 0.9, >0.32, 2.2 and 58 mg/L, respectively [52]. Even though the concentrations of some endocrine disruptors determined in the river water were lower than LC50 values of these chemicals, many other endocrine disruptors coexisted in the surface water, which also induced endocrine disrupt effects. High estrogenic activities were measured in the surface water from the Hun River using in vitro bioassay [53]. Hence, the comparative analysis of the transcriptomic profile of the complex environmental samples with the model compound, may prospectively enable the identification of the pollutants based on toxic mechanism, providing a powerful tool for effect-directed analysis.

## 5. Conclusions

In summary, the present study evaluates the feasibility of using hepatic transcriptomic profiling in zebrafish in the effect-directed monitoring of surface water. While changes in gene expression do not always correlate to changes in metabolism, the DEGs number and the pathway enrichment are indicative of the pollution gradient. The gene expression profiles suggested endocrine disruption effects and genotoxic effects, and potential immunotoxic effects and disruption of circulation system. The pathways related with human endocrine and metabolic diseases, and infectious diseases, were also affected by the surface water. The transcriptomic profile differentiated the sampling sites from the tributary and mainstream, and upstream and downstream of Shenyang. The comparative analysis of the transcriptomic profile of the complex surface water with that of model compounds suggested relatively higher similarity between surface water and E2, than other several organic and metal compounds, exhibiting the potential to identify the pollutants patterns based on toxic mechanism. The development of the cross-platform read of the transcriptomic data, and the annotation of the currently unknown genes, as well as measuring the activity of enzymes whose gene expression is affected by aquatic effluents would profit the genomic tools to be more powerful in prediction of toxic effects and pollutants identification in surface water.

## Figures and Tables

**Table 1:** Sampling location and physicochemical water parameters.

Site	Longitude (East)	Latitude (North)	pH	Dissolved Oxygen (mg/L)	Chemical Oxygen Demand (mg/L)	Ammonia Nitrogen (mg/L)
**H1**	124°05.627	41°53.426	8.41	7.89	7	0.1
**H2**	123°58.214	41°52.640	7.06	8.01	5	0.1
**H3**	123°34.452	41°48.704	7.01	5.03	9	2.3
**H4**	123°18.311	41°42.900	7.91	9.57	13	2.7
**H5**	122°57.322	41°29.674	7.94	7.45	2	0.1
**X**	122°59.705	41°31.554	7.75	6.30	10	0.1
**D**	124°02.061	41°51.695	7.32	7.53	20	0.03

**Table 2:** Concentrations of organic chemicals in surface water samples.

Chemicals	Concentration (μg/L)				
	H1	H2	H3	H4	D
Diisobutyl phthalate	5.03	35.5	80.2	20.2	125
Di(2-ethylhexyl) phthalate	1.39	3.43	9.71	4.36	11.3
2,4-di-*tert*-butylphenol	1.59	2.54	7.31	4.65	8.87
Benzyl benzoate	6.12	6.49	6.54	6.42	7.26
*m*-Cresol	1.91	1.23	1.51	1.42	5.67
Dibutyl phthalate	0.975	2.12	2.36	2.62	3.36
2-Methylpyridine	1.7	1.42	1.05	1.04	2.55
2,6-di-*tert*-butyl-*p*-cresol	0.558	1.018	2.22	0.865	1.92
*o*-Cresol	0.821	0.48	0.536	0.449	1.522
2-Methylnaphthalene	0.568	0.471	0.787	0.512	1.201
Dimethyl phthalate	0.585	0.855	0.813	1.77	1.06

## Supplementary Materials

The following are available online at http://www.mdpi.com/1660-4601/15/8/1648/s1. Figure S1. Evaluation of gene expression across pollution gradients and discharge source in the Hun River. Figure S2. Clustering analysis of gene expression of zebrafish exposed to the Hun River water from each site with exposed to selected individual chemicals. Table S1. Number of DEGs with fold change ≥ 2 in the livers of zebrafish exposed to surface water. Table S2. Pearson correlations between number of DEGs and physicochemical parameters. Table S3. Concentrations of organic chemicals in surface water samples.
