# Supplementary material for: Transcriptomic Profiles in Zebrafish Liver Permit the Discrimination of Surface Water with Pollution Gradient and Different Discharges
Source: Int J Environ Res Public Health. 2018 Aug 3;15(8):1648. doi: 10.3390/ijerph15081648 (PMC6122030; doi:10.3390/ijerph15081648)
Supplement: Supplementary file 1 [file ijerph-15-01648-s001.pdf]

Support information

Transcriptomic Profiles in Zebrafish Liver Permit the Discrimination of Surface Water with Pollution Gradient and Different Discharges

Zhou Zhang [1], Wei Liu [1,*,†], Yuanyuan Qu [1], Xie Quan [1], Ping Zeng [2], Mengchang He [3], Yanmei Zhou [4,†] and Ruixia Liu [2,†]

[1] Key Laboratory of Industrial Ecology and Environmental Engineering (MOE), School of Environmental Science and Technology, Dalian University of Technology, Dalian 116024, China; andar2008@126.com (Z.Z.); qyy007@126.com (Y.Q.); quanxie@dlut.edu.cn (X.Q.)
[2] State Key Laboratory of Environmental Criteria and Risk Assessment, Chinese Research Academy of Environmental Science, Beijing 100012, China; zengping@craes.org.cn (P.Z.); liurx@craes.org.cn (R.L.)
[3] State Key Laboratory of Water Environment Simulation, School of Environment, Beijing Normal University, Beijing 100875, China; hemc@bnu.edu.cn
[4] Department of Civil and Environmental Engineering, Beijing Key Laboratory of Aqueous Typical Pollutants Control and Water Quality Safeguard, Beijing Jiaotong University, Beijing 100044, China; zym721101@163.com
* Correspondence: liu_wei@dlut.edu.cn
† These authors contributed equally to this work

Methods of chemical analysis

Organic chemicals (such as phthalic acid esters (PAEs), polycyclic aromatic hydrocarbons (PAHs), benzene hydrocarbons and phenolic compounds) in water samples were quantitatively analyzed by gas chromatography-mass spectrography (GC-MS, 463 GC-scion MS, Bruker, Germany). 50 μL 1mg/L phenanthrene-D10 and acenaphthylene-D10 was added to 5L water samples as surrogates to quantify recoveries. Samples were extracted using polar (Oasis, HLB, Waters Co., USA) and nonpolar solid phase extraction columns (Supelclean ENVI – 81 SPE, Supelco, USA) successively activated by 10 mL acetone, 10 mL acetone:methanol (9:1, v/v) (Mreda Technology Inc., USA) and 10 mL high purity water (Millipore, USA) and connected in series. Two columns were then eluted applying 10 mL acetone:methanol (9:1, v/v) and 10mL acetone:hexane (3:7, v/v) (Mreda Technology Inc., USA), respectively. Extracts were concentrated by pressured nitrogen blowing concentrators and analyzed by GC-MS using anthracene-D10 as internal standard. GC was equipped with a capillary column (DB-5MS, 30 m × 0.25 mm × 0.25 μm, Agilent Co.) and sample size was 1.0 μL (non flow injection). Carrier gas was He (purity 99.99%) and flow rate was 1 mL/min. The temperature of introduction port was 280 °C and detector was 300°C. The start temperature of column was 40°C, stayed for 2 min, temperature programmed up to 300°C by the rate of 5°C /min and then stayed for 5min. SIM scan was selected. The determination condition of MS was: ion source (EI) temperature of 270°C, quadrupole temperature of 200°C and EI voltage of 70eV.

To avoid the contamination, the plastic ware was excluded and the pretreatment of all samples were carried out in a super-clean work bench. Additionally, the blank contamination control experiment was performed in the same manner as the samples to determine any background contamination. The concentration of these pollutants in the samples was blank corrected. Three matrix samples spiked with mixed standards at the two levels of 500 and 1000 ng/L were run to monitor the recoveries of the analytical method. Surrogate recoveries for all samples were from 77% to 113% for both phenanthrene-D10 and acenaphthylene-D10. The limit of detection (LOD) of the analytes was determined with a signal-to-noise ratio of 3:1, while the limit of quantification (LOQ) was determined with a signal-to-noise ratio of 10:1. The concentrations of organic compounds were calculated using standard curves. Standard curves of the target chemicals were prepared by

increasing the concentration of contaminants from 20 to 1000 μg/L at six level and spiking the fixed levels (100 μg/L) of the internal standards, with correlation of coefficients more than 0.99.

Table S1. Number of DEGs with fold change ≥ 2 in the livers of zebrafish exposed to surface water.

Sample	Total	Up-	Down-
H1	2275	820	1455
H2	728	206	522
H3	2648	1032	1616
H4	1685	1043	642
H5	885	487	398
X	2046	744	1302
D *	3292	2018	1274

* DEGs of Site D were FC ≥ 3.

Table S2. Pearson correlations between number of DEGs and physicochemical parameters.

	DEGs	COD	NH_3-N	DO	pH
**DEGs**	1	0.788*	0.124	-0.355	-0.102
**COD**		1	0.183	0.105	-0.199
**NH_3-N**			1	0.043	-0.165
**DO**				1	0.443
**pH**					1

*. Correlation is significant at the 0.05 level.

Table S3. Concentrations of organic chemicals in surface water samples.

Chemicals	Concentration (μg/l)				
	H1	H2	H3	H4	D
n-Propylbenzene	0.358	0.438	0.169	0.447	0.312
m-Ethyltoluene	0.333	0.428	0.088	0.299	0.329
Mesitylene	0.043	0.077	0.023	0.066	0.061
2-Ethyltoluene	0.017	0.023	0.004	0.026	0.017
1,2,4-Trimethylbenzene	0.061	0.078	0.028	0.029	0.058
4-Methylstyrene	ND	0.013	0.019	0.157	0.010
1,2,3-Trimethylbenzene	0.488	0.661	0.233	0.608	0.501
1,3-Diethylbenzene	0.173	0.187	0.085	0.274	0.144
1,4-Diethylbenzene	0.101	0.143	0.039	0.167	0.112
1,2-Diethylbenzene	0.114	0.198	0.091	0.184	0.139
1,2,4,5-tetramethylbenzene	0.089	0.122	0.079	0.107	0.086
1,2,3,4-tetramethylbenzene	0.123	0.176	0.117	0.265	0.138
Pentamethylbenzene	0.118	0.172	0.229	0.208	0.134
Hexamethylbenzene	ND	ND	ND	0.012	ND
Naphthalene	0.230	0.162	0.230	0.218	0.541
2-Methylnaphthalene	0.568	0.471	0.787	0.512	1.201
1-Methylnaphthalene	0.333	0.321	0.615	0.351	0.739
2,7-Dimethylnaphthalene	0.128	0.116	0.469	0.149	0.362
1,3-Dimethylnaphthalene	0.099	0.099	0.549	0.161	0.324
1,4-Dimethylnaphthalene	0.061	0.071	0.420	0.118	0.182
Acenaphthylene	0.340	0.236	0.316	0.265	0.660
Acenaphthene	0.010	0.012	0.045	0.058	0.023
Fluorene	0.069	0.053	0.050	0.059	0.122
Phenanthrene	0.005	0.007	0.022	0.019	0.013
2-Methylphenanthrene	0.018	0.037	0.381	0.061	0.085

1-Methylanthracene	0.049	0.109	1.228	0.265	0.157
Fluoranthene	0.012	0.016	0.124	0.062	0.034
Pyrene	0.007	0.018	0.257	0.051	0.020
1-Methylpyrene	0.005	0.015	0.393	0.01	0.013
Triphenylene	0.016	0.022	0.433	0.043	0.024
Benz(a)anthracene	0.016	0.026	0.387	0.044	0.036
Chrysene	0.002	0.003	0.007	0.003	0.003
Benzo(b)fluoranthene	0.005	0.004	0.008	0.008	0.015
o-Cresol	0.821	0.48	0.536	0.449	1.522
m-Cresol	1.91	1.23	1.51	1.42	5.67
Guaiacol	0.322	0.115	0.332	0.367	0.585
2,6-Dimethylphenol	0.077	0.111	0.265	0.123	0.175
2-Ethylphenol	0.049	0.041	0.061	0.040	0.144
2,4-Dimethylphenol	0.415	0.194	0.349	0.268	0.943
2,5-Dimethylphenol	0.427	0.324	0.648	0.207	1.058
4-Ethylphenol	0.240	0.157	0.311	0.156	0.725
3-Ethylphenol	0.356	0.270	0.381	0.268	0.913
3,5-Dimethylphenol	0.239	0.129	0.373	0.113	0.882
3,4-Dimethylphenol	0.177	0.138	0.330	0.112	0.809
2,4,6-trimethylphenol	0.028	0.023	0.101	0.026	0.061
4-Methoxyphenol	0.091	0.055	0.080	0.143	0.366
2-n-propylphenol	0.014	0.023	0.075	0.079	0.030
2,3,6-Trimethylphenol	0.016	0.024	0.092	0.033	0.055
4-Methyl-2-nitrophenol	0.101	0.178	0.487	0.549	0.366
5-Methyl-2-nitrophenol	0.026	0.084	0.061	0.029	ND
2,3,5-trimethylphenol	0.029	0.027	0.062	0.035	0.107
3,4,5-trimethylphenol	ND	ND	ND	ND	0.071
2,4-di-tert-butylphenol	1.59	2.54	7.31	4.65	8.87
2,6-di-tert-butyl-p-cresol	0.558	1.018	2.22	0.865	1.92
3-Cyanopyridine	0.023	0.021	0.039	0.064	0.040
2-Methylglutaronitrile	0.370	0.122	0.059	0.233	0.145
1,3-Dicyanobenzene	0.019	0.020	0.039	0.027	0.038
Benzaldehyde	0.111	0.018	0.092	0.081	0.056
2-Ethylhexanol	0.388	0.368	0.408	0.262	0.421
Acetophenone	0.494	0.606	0.369	0.512	0.528
1,2,3,4-Tetrahydronaphthalene	0.036	0.029	0.065	0.038	0.064
1-Tetralone	ND	0.018	0.084	0.056	0.023
Benzyl benzoate	6.12	6.49	6.54	6.42	7.26
3,5-di-tert-butyl-4-hydroxybenzaldehyde	0.223	0.394	0.704	0.786	0.684
2-Naphthylamine	0.044	ND	0.017	0.012	ND
1,4-Dinitrobenzene	0.216	0.223	0.664	0.283	0.548
Amino biphenyl	0.165	ND	0.204	0.075	ND
2-Methylpyridine	1.70	1.42	1.05	1.04	2.55
2,6-lutidine	0.249	0.224	0.230	0.275	0.449
Indene	0.214	0.184	0.281	0.173	0.436
1-Indanone	0.071	0.129	0.575	0.386	0.130
2-Methylquinoline	0.022	0.017	0.080	0.117	ND
2-Methylindole	ND	ND	ND	ND	0.024
1H-Benzotriazole	ND	ND	2.14	0.622	ND
7,8-Benzoquinoline	0.024	0.037	0.130	0.082	0.072
Acridine	ND	ND	0.018	ND	ND
Dimethyl phthalate	0.585	0.855	0.813	1.77	1.06

Diethyl phthalate	0.238	0.398	0.538	0.816	0.525
Diisobutyl phthalate	5.03	35.5	80.2	20.2	125
Dibutyl phthalate	0.975	2.12	2.36	2.62	3.36
Butylbenzyl phthalate	0.016	0.016	0.015	0.011	0.019
Di(2-ethylhexyl) phthalate	1.39	3.43	9.71	4.36	11.3

ND: not detected.

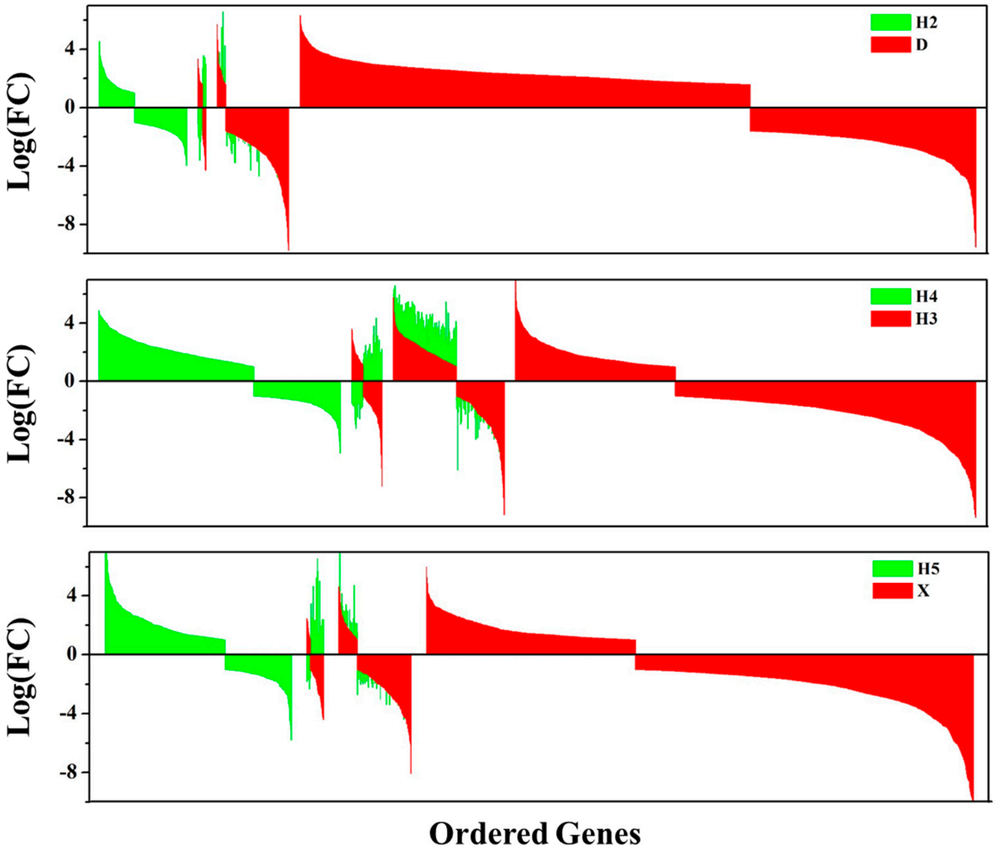

Figure S1. Evaluation of gene expression across pollution gradients and discharge source in the Hun River. The four groups consisted of unique gene responses at downstream and upstream (tributary), and genes with the same response and genes with the opposite regulation.

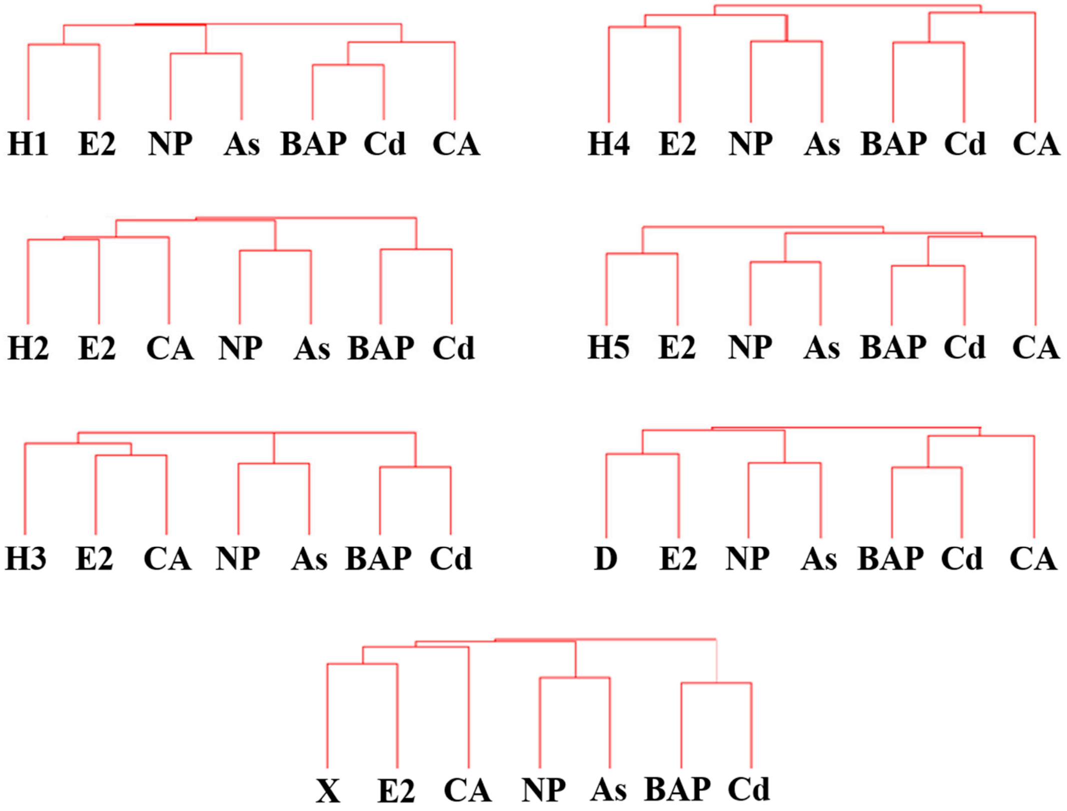

Figure S2. Clustering analysis of gene expression of zebrafish exposed to the Hun River water from each site with exposed to selected individual chemicals. E2: estradiol, NP: nitrophenol, As: arsenic, BAP: benzo-[A]-pyrene,Cd: cadmium, CA: chloroaniline.

© 2018 by the authors. Submitted for possible open access publication under the terms and conditions of the Creative Commons Attribution (CC BY) license (http://creativecommons.org/licenses/by/4.0/).
